# Excision repair cross-complementing group 1 (ERCC1) overexpression inhibits cell apoptosis and is associated with unfavorable prognosis of esophageal squamous cell carcinoma: Erratum

**DOI:** 10.1097/MD.0000000000012163

**Published:** 2018-08-24

In the article, “Excision repair cross-complementing group 1 (ERCC1) overexpression inhibits cell apoptosis and is associated with unfavorable prognosis of esophageal squamous cell carcinoma”,^[1]^ which appeared in Volume 97, Issue 31 of *Medicine*, the authors would like to add the funding information “This work was supported by Shandong Medicine and Health Science Technology Development Plan 2014WS0288.”
